# Hmgcs2 is the hub gene in diabetic cardiomyopathy and is negatively regulated by Hmgcs2, promoting high glucose‐induced cardiomyocyte injury

**DOI:** 10.1002/iid3.1191

**Published:** 2024-03-13

**Authors:** Ying Wang, Li‐Feng Ping, Fu‐Yan Bai, Xin‐Huan Zhang, Guang‐Hong Li

**Affiliations:** ^1^ Department of Endocrinology The Second Affiliated Hospital of Shandong First Medical University Tai'an China; ^2^ Department of General Medicine The Second Affiliated Hospital of Shandong First Medical University Tai'an China

**Keywords:** diabetic cardiomyopathy, GEO database chip, high glucose, hmgcs2, miR‐363‐5p

## Abstract

**Background:**

Diabetic cardiomyopathy (DCM) represents a major cause of heart failure and a large medical burden worldwide. This study screened the potentially regulatory targets of DCM and analyzed their roles in high glucose (HG)‐induced cardiomyocyte injury.

**Methods:**

Through GEO database, we obtained rat DCM expression chips and screened differentially expressed genes. Rat cardiomyocytes (H9C2) were induced with HG. 3‐hydroxy‐3‐methylglutarylcoenzyme A synthase 2 (Hmgcs2) and microRNA (miR)‐363‐5p expression patterns in cells were measured by real‐time quantitative polymerase chain reaction or Western blot assay, with the dual‐luciferase assay to analyze their binding relationship. Then, 3‐(4,5‐dimethyl‐2‐thiazolyl)‐2,5‐diphenyl‐2‐H‐tetrazolium bromide assay, lactate dehydrogenase assay, terminal deoxynucleotidyl transferase dUTP nick end labeling assay, enzyme‐linked immunosorbent assay, and various assay kits were applied to evaluate cell viability, cytotoxicity, apoptosis, inflammation responses, and oxidative burden.

**Results:**

Hmgcs2 was the vital hub gene in DCM. Hmgcs2 was upregulated in HG‐induced cardiomyocytes. Hmgcs2 downregulation increased cell viability, decreased TUNEL‐positive cell number, reduced HG‐induced inflammation and oxidative stress. miR‐363‐5p is the upstream miRNA of Hmgcs2. miR‐363‐5p overexpression attenuated HG‐induced cell injury.

**Conclusions:**

Hmgcs2 had the most critical regulatory role in DCM. We for the first time reported that miR‐363‐5p inhibited Hmgcs2 expression, thereby alleviating HG‐induced cardiomyocyte injury.

## Graphical abstract

Diabetic cardiomyopathy (DCM) represents a major cause of heart failure and a large medical burden worldwide. This study screened the potentially regulatory targets of DCM and analyzed their roles in high glucose ‐induced cardiomyocyte injury.

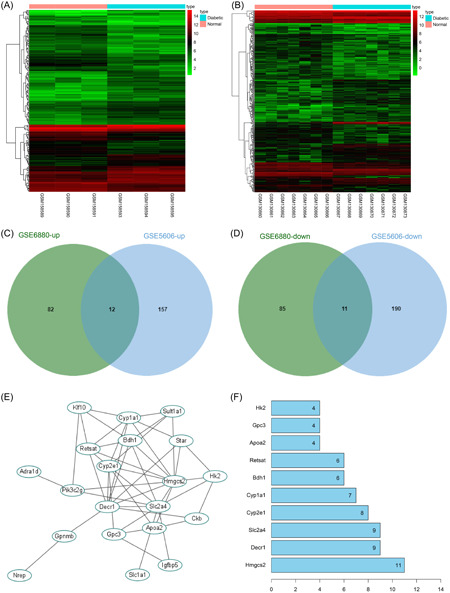

## 1 INTRODUCTION

Longstanding diabetes mellitus (DM) is inclined to induce a decline of cardiac functions in absence of all other cardiovascular diseases, which is an event defined as diabetic cardiomyopathy (DCM).^1^ DCM is a major cause of heart failure and constitutes a large medical burden worldwide, however, its pathophysiology remains not well understood.^2^ Hyperglycemia is a principal trigger factor of DCM pathogenesis,^3^ and high glucose (HG) is a commonly used method to induce in vitro DCM model.^4^, ^5^ Specifically, HG disrupts the integrity of mitochondria together with reactive oxygen species (ROS) and nitrogen overproduction, leading to oxidative injury in heart tissue.^6^ Meanwhile, increases in myocardial intercellular adhesion molecule 1 and vascular cell adhesion molecule 1 levels occur with the release of pro‐inflammatory cytokine like tumor necrosis factor alpha (TNF‐α) and interleukin 1 beta (IL‐1β) from infiltrating immune cells, contributing to immune dysfunction, intramyocardial inflammation, and cardiomyocyte death.^7^ Interestingly, increased oxidative stress and inflammation are positively correlated with impairment of left ventricular (LV) function in diabetic heart.^8^ Therefore, it is essential to decipher the molecular route of HG‐induced inflammation and oxidative stress, thus providing novel targets for DCM treatment.

High‐throughput sequencing is an effective approach to explore the molecular mechanism underlying DCM and identify relevant biomarkers. For instance, a microarray profiling study showed that LDHA, ALDOC, ABCE1, ATF4 and MYH6 regulate the metabolic process and cell cycle in DCM^9^; using high‑throughput RNA sequencing technology, 58 significantly differentially expressed circular RNAs are shown to correlate with early‑stage DCM.^10^ Of note, 3‐hydroxy‐3‐methylglutarylcoenzyme A synthase 2 (Hmgcs2) is a rate‐limiting enzyme of ketogenesis.^11^ The diabetic heart has been shown increased ketogenesis and ketogenic enzymes levels, including Hmgcs2.^12^ Additionally, Hmgcs2 knockdown is shown to alleviate HG‐induced myocardial injury.^13^ In our study, we employed microarray datasets (GSE560 and GSE6880 expression chips) to screen differentially expressed genes (DEGs) and conducted gene interaction analysis to identify hub genes. As a result, Hmgcs2 was uncovered as a hub gene in DCM and was further tested by cellular functional experiments to validate its role.

microRNAs (miRNAs) are known as a class of single‐strand noncoding RNAs that can degrade the message RNA (mRNA) by matching with the 3′UTR of mRNA.^14^ miRNAs play roles in cardiac regeneration and cardiac functions and can serve as therapeutic targets for DCM.^15^ Our bioinformatic prediction of miRNA showed that miR‐363‐5p was a potential upstream miRNA of Hmgcs2. Accumulated evidence have suggested that miR‐363‐5p is of importance for immunomodulation, endothelial function, neurite outgrowth, anti‐tumorigenesis.^16^, ^17^, ^18^, ^19^ Intriguingly, miR‐363 can regulate cardiomyocyte loss and LV development.^20^, ^21^ In the same light, we hypothesized that miR‐363‐5p may participate in the development of DCM by targeting Hmgcs2. The current study aimed to evaluate the role of the miR‐363‐5p/Hmgcs2 axis in HG‐induced cardiomyocyte injury, reporting the upstream mechanism of Hmgcs2 for the first time and providing additional evidence for the role of Hmgcs2 in DCM.

## 2 METHODS

### 2.1 Bioinformatic analysis

The mRNA expression chips of DCM mice, GSE5606 and GSE6880, were obtained from Gene Expression Omnibus (GEO) database (https://www.ncbi.nlm.nih.gov/geo/). With the normal specimens as the control, differential analysis was performed using limma package of R language. |logFC | >1 and *p* < .05 were used as screening standards to obtain significant DEGs in DCM. Subsequently, the intersections were respectively taken from the upregulated and downregulated genes showed by two expression chips. The candidate genes in intersections were subjected to gene interaction analysis, and a map of gene interaction network was plotted. The degree value of each gene in the network map was calculated to obtain DCM‐related hub genes. The upstream miRNAs of candidate genes were predicted using TargetScan database (https://www.targetscan.org/vert_71/)^22^ and miRDB database (https://mirdb.org/index.html),^23^ and the intersection of predicted genes was identified.

### 2.2 Cell culture and treatment

Rat cardiomyocytes (H9C2 cell lineage) were procured from ATCC corporation. H9C2 cells were pre‐cultured in the conventional conditions (Dulbecco's modified Eagle medium [DMEM; Gibco, BRL], 10% fetal bovine serum [Sigma], a humidified air, 37°C, 5% CO_2_). After that, H9C2 cells were cultured in DMEM containing 30 mmol/L glucose for 24 h to establish the in vitro DCM model,^24^ with cells cultured in DMEM containing 5.5 mmol/L glucose as the control group. Specific small interfering RNAs targeting Hmgcs2 (si‐Hmgcs2#1, si‐Hmgcs2#2) and si‐NC were bought from GenePharma corporation, and miR‐363‐5p mimics, and mimic NC were provided by RiboBio. According to the protocol, the above siRNAs or sequences were transfected into H9C2 cells using Lipofectamine 2000 (Invitrogen). The subsequent experiments were conducted at 48 h after transfection.

### 2.3 Cell viability

Cell viability was determined by the 3‐(4,5‐dimethylthiazol‐2‐yl)‐2,5‐diphenyltetrazolium bromide (MTT) method. H9C2 cells were grown in a 96‐well plate for 24 h at a density of 1 × 10^4^ cells/well. At a final concentration of 0.5 mg/mL, MTT was incorporated into H9C2 cells with different treatments. After 4 h, each well was incorporated with 100 μL dimethylsulfoxide, with a microplate reader (Thermo Fisher Scientific) with 490 nm wavelength to determine absorbance. Cell viability was presented as the ratio of optical density (OD) of sample to OD of control.

### 2.4 Lactate dehydrogenase (LDH) assay

The release of LDH from H9C2 cells was determined using a LDH kit (Beyotime). Liquid supernatant (50 μL) of H9C2 cells was selected from every well, followed by 30 min incubation with reduced nicotinamide adenine dinucleotide and pyruvic acid at 37°C. After 15 min, 0.4 mol/L NaOH was added to terminate the reaction. Sample absorbance at 440 nm wavelength was measured using a microplate reader and was converted into LDH activity.

### 2.5 Terminal‐deoxynucleoitidyl transferase mediated Nick end labeling (TUNEL) assay

The apoptosis rate of H9C2 cells was determined by means of TUNEL staining using a in situ cell death detection kit (Roche Molecular Biochemicals). Briefly, H9C2 cells with different treatments were fixed with 4% paraformaldehyde and permeated with 0.1% Triton X‐100. Next, TUNEL staining was conducted in accordance with the producer's instructions, followed by staining with 4′,6‐diamidino‐2‐phenylindole. The apoptosis rate was the ratio of TUNEL‐positive cell number to the total cell number.

### 2.6 Oxidative stress injury assay

The activity of superoxide dismutase (SOD) in H9C2 cells was determined using a SOD activity determination kit (S0109, Beyotime). Simply put, H9C2 cells with different treatments were lysed and tested by the kit, followed by determination of absorbance at 505 nm wavelength.

The content of malondialdehyde (MDA) in H9C2 was determined using an MDA assay kit (S0131M, Beyotime). Simply put, H9C2 cells with different treatments were homogenized and mixed with thiobarbituric acid reagent. Subsequently, the cocktail was boiled for 15 min and was centrifuged at 4000 g for 8 min, followed by determination of absorbance at 532 nm wavelength.

The amount of ROS was measured using a fluorescence probe redox‐sensitive‐fluoroprobe; 2′,7′‐dichlorofluorescein‐diacetate (DCFDA; 287810, Sigma) and a ROS detection kit (88‐5930‐74, Invitrogen). DMEM (1: 1000) was used to dilute DCFDA to the ultimate concentration of 10 μM. After removal of culture medium, diluted DCFDA was added until the complete coverage of H9C2 cells, followed by 1 h culture at 37°C in the dark. Excess DCFDA was removed by 3 washes with phosphate buffered saline, upon which fluorescence intensity was evaluated with a laser confocal microscope (FV1000, Olympus, excitation wavelength: 480 nm, emission wavelength: 530 nm) to present the relative ROS levels in H9C2 cells.

### 2.7 Enzyme‐linked immunosorbent assay

H9C2 cells were cultured in a 6‐well plate for 72 h. After that, the supernatant was collected to determine the amounts of tumor necrosis factor (TNF)‐α (ab236712), interleukin (IL)‐6 (ab100772), and IL‐1β (ab255730) by employing ELISA kits (Abcam).

### 2.8 Dual‐luciferase assay

The potential binding site of miR‐363‐5p and Hmgcs2 was predicted by the Targetscan database. According to the prediction results, the wildtype (WT) and mutant type (MUT) sequences containing the binding site of miR‐363‐5p and Hmgcs2 were designed respectively. WT or MUT sequence was inserted into luciferase reporter vectors (pGL3‐Basic; Promega, Madison, WI, USA) to generate the vectors WT‐Hmgcs2 and MUT‐Hmgcs2. Subsequently, WT‐Hmgcs2 or MUT‐Hmgcs2 was cotransfected with miR‐363‐5p mimics or mimics NC into H9C2 cells using a Lipofectamine 2000 kit. Around 48 h after transfection, the luciferase activity was measured with the aid of dual‐luciferase reporter analysis system (Promega).

### 2.9 Real‐time quantitative polymerase chain reaction (RT‐qPCR)

RNA was separated from H9C2 cells with use of the TRIzol reagent (Invitrogen), with RNase‐free DNase (Ambion) to eliminate genomic DNA contamination and Super‐Script II (Invitrogen) to convert RNA into the complementary DNA. qPCR ran on with the support of SYBR Green Master Mix (Roche, Shanghai, China) and CFX97 system (Bio‐Rad). With glyceraldehyde‐3‐phosphate dehydrogenase (GAPDH) and U6^25^ serving as control genes, the relative amount of gene expression was calculated based on the 2^‐ΔΔCt^ method.^26^ Sequence information of primers are listed in Table 1.

**Table 1:** PCR primers sequences.

Gene	Sequence (5′‐3′)
Hmgcs2	F: GGCTGATGGAACGCACAAAG
	R: CCTCGATGTCAGTGTTGCCT
GAPDH	F: ACGGGAAACCCATCACCATC
	R: ACGACATACTCAGCACCAGC
miR‐363‐5p	F: ATCTACGCGGGTGGATCACGA
	R: CAACTGGTGTCGTGGAGTCGG
U6	F: ATGGCGGACGACGTAGATCA
	R: TCAGCCAACTCTCAATGGAGG

### 2.10 Western blot assay

The radioimmunoprecipitation assay buffer was used to extract the total protein from H9C2 cells and the bicinchoninic acid method was used for protein quantification. The supernatant of cell extracts was separated on 10% sodium dodecyl sulfate polyacrylamide gel electrophoresis, followed by transference onto polyvinylidene fluoride membranes. The membranes were blockaded with Tween 20 (TBST; 50 mM Tris, 150 mM NaCl, 0.1% Tween 20, pH 7.6) and Tris‐buffered saline containing 5% skim milk at ambient temperature for 2 h, followed by overnight incubation with antibodies against Hmgcs2 (ab137043, 1:1000, Abcam) and GAPDH (ab181602, 1:10000) at 4°C. Next, the blots were washed thrice with TBST (Solarbio, Beijing, China), followed by 2 h incubation with the secondary antibody (ab205718, 1:2000, Abcam) at ambient temperature. The grayscale value was analyzed with the use of NIH Image J software (National Institutes of Health).

### 2.11 Statistical analysis

Data statistical analysis and graphing were conducted with the use of SPSS21.0 statistical software (IBM SPSS Statistics) and GraphPad Prism 8.0 software (GraphPad Software Inc.). Data were tested to be normally distributed and homogeneous in variances. The pairwise comparisons of measurement data were analyzed by the *t*‐test, multi‐group comparisons were analyzed by one‐way or two‐way analysis of variance (ANOVA), with Tukey's multiple comparison test upon ANOVA. *P* values were attained from the two‐sided test. *p* < .05 was considered differences with statistical significance and *p* < .01 was considered differences with high statistical significance.

## 3 RESULTS

### 3.1 Identification of DEGs in GSE6880 and GSE5606

**Figure 1:**
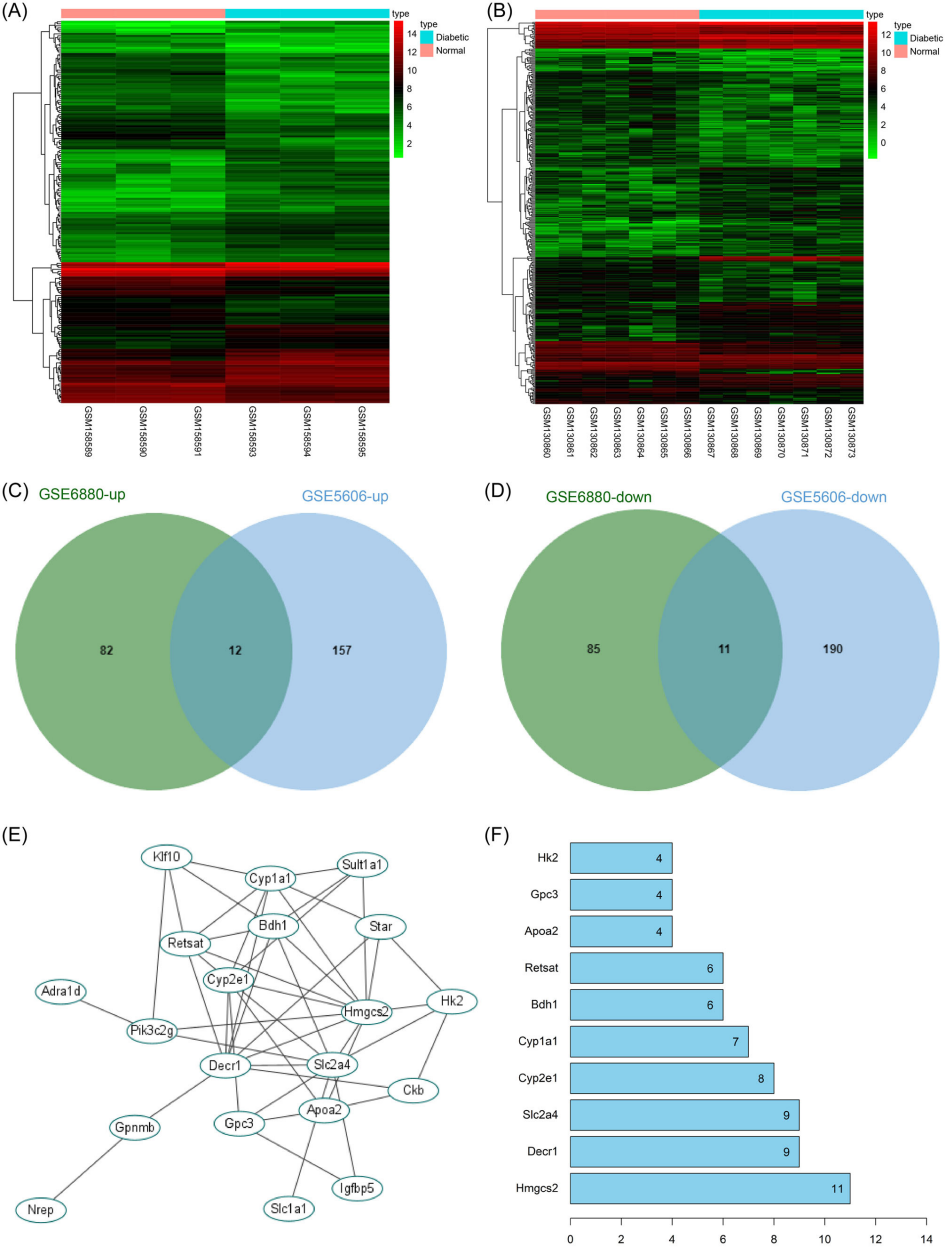
Identification of differentially expressed genes in GSE6880 and GSE5606. (A, B) Heat maps of differentially expressed genes in GSE6880 and GSE5606, the x‐coordinate represents sample number, tree diagram, and the upper right bars represent color gradation. (C, D) Intersections of upregulated and downregulated DEGs in two chips. (E) Map of candidate gene interaction network, each ellipse represents a gene, and lines between genes indicate interactions between genes. (F) The top 10 genes with the highest degree value in gene interaction network map, the x‐coordinate represents degree value, the greater degree value of the gene indicates it has more interacting genes and it is more core.

Genes in rat DCM expression chips GSE6880 and GSE5606 were subjected to differential analysis, resulting in identification of 190 significant DEGs from GSE6880 (Figure 1A) and 370 significant DEGs from GSE5606 (Figure 1B). The intersections of significantly upregulated DEGs and significantly downregulated DEGs obtained from two chips were identified respectively, which revealed the presence of 12 upregulated genes (Figure 1C) and 11 downregulated genes (Figure 1D). The gene interaction analysis was performed on the 23 candidate genes (Figure 1E), and the gene interaction network map was constructed to calculate the degree value of each gene in the map. It was found that Hmgcs2 had the highest degree value (Figure 1F), suggesting that Hmgcs2 had the most critical regulatory role in DCM among these genes. Therefore, we selected Hmgcs2 for further study to validate its role.

### 3.2 Hmgcs2 downregulation inhibits HG‐induced cell injury

**Figure 2:**
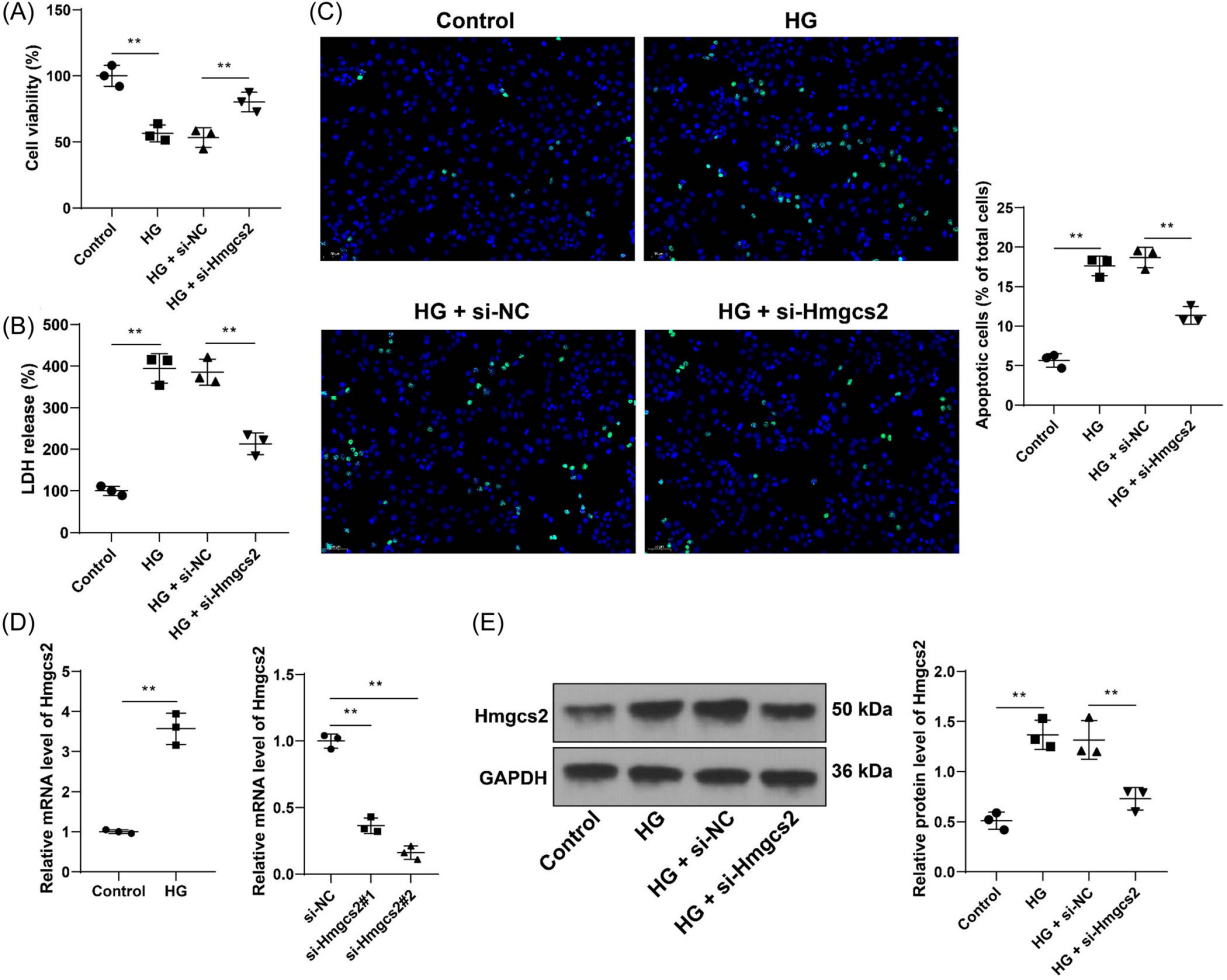
Hmgcs2 downregulation inhibits HG‐induced cell injury. H9C2 cells were transfected with Hmgcs2 siRNA (si‐Hmgcs2), with cells transfected with si‐NC as the negative control, followed by high glucose induction. (A) Cell viability was examined by MTT assay. (B) LDH release of each cell group. (C) Cell apoptosis was assessed by TUNEL assay (scale bar = 50 μm). (D) mRNA levels of Hmgcs2 were determined by RT‐qPCR. (E) Expression levels of Hmgcs2 were determined by Western blot assay. Cell experiments were performed in triplicate. *N* = 3. Data in panel D (left) were analyzed by the *t* test, and data in panels A–E were analyzed by one‐way ANOVA, followed by Tukey's multiple comparison test. **p < .01.

H9C2 cells were induced with HG to establish the in vitro DCM model, upon which cell viability was decreased and LDH release and apoptosis rate was increased (*p* < .01, Figure 2A–C). HG induction increased the expression levels of Hmgcs2 (*p* < .01, Figure 2D,E). Next, H9C2 cells were transfected with Hmgcs2 siRNA (si‐Hmgcs2) to downregulate intracellular expression of Hmgcs2 (*p* < .01, Figure 2D,E). si‐Hmgcs2#2 was found to have the higher silencing efficiency and as a result was selected for the subsequent assays. Our results showed that after downregulation of Hmgcs2, cell viability was elevated and LDH release and apoptosis rate was reduced (*p* < .01, Figure 2A–C). These results suggested that Hmgcs2 downregulation inhibited HG‐induced cell injury.

### 3.3 Hmgcs2 downregulation inhibits HG‐induced cell inflammation and oxidative stress

**Figure 3:**
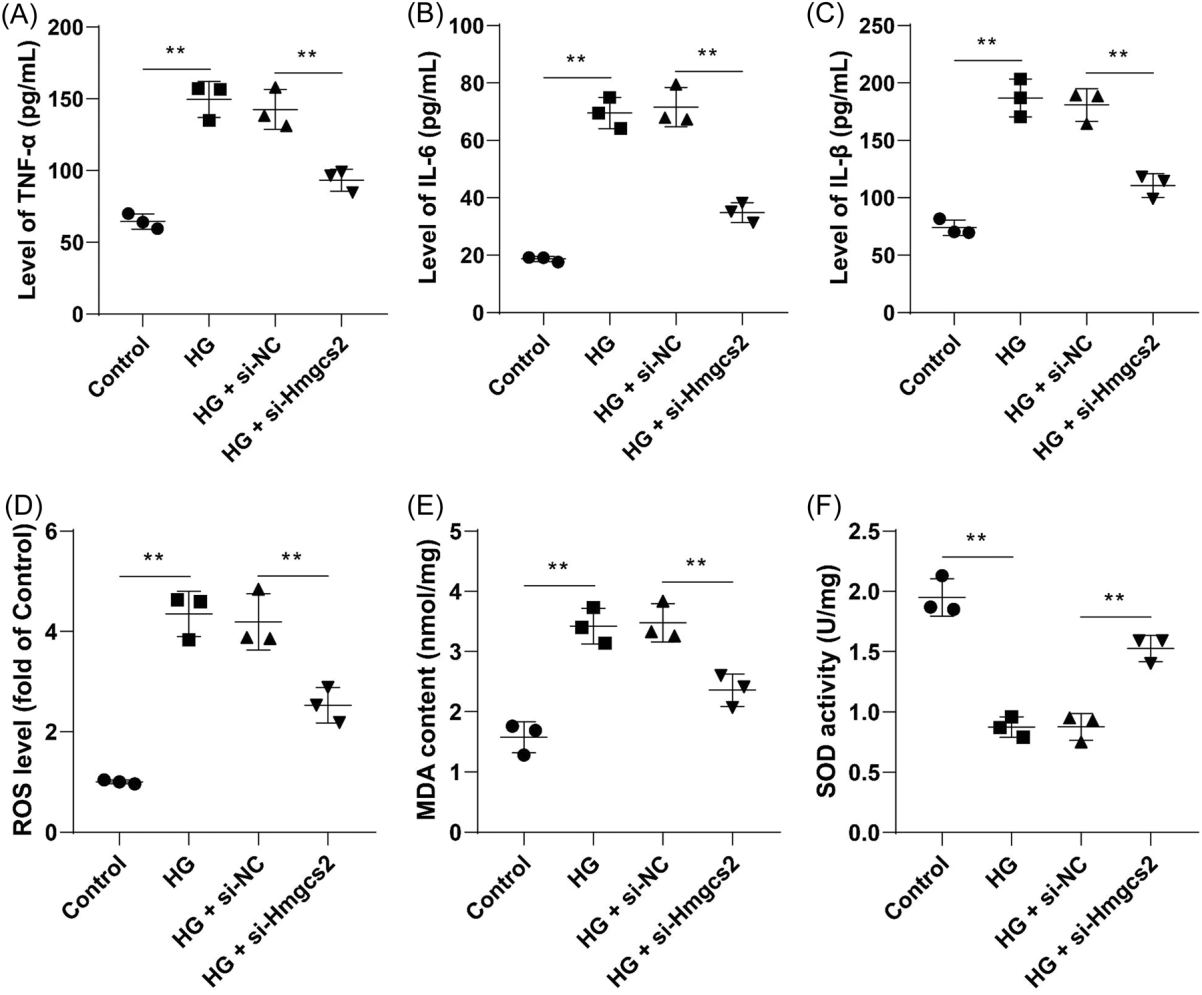
Hmgcs2 downregulation inhibits HG‐induced cell inflammation and oxidative stress. (A–C) Contents of TNF‐α, IL‐6, and IL‐1β were determined by ELISA. (D) ROS levels of each cell group; E: MDA levels of each cell group. (F) SOD levels of each cell group. *N* = 3. Data in panels A–F were analyzed by one‐way ANOVA, followed by Tukey's multiple comparisons test. **p < .01.

By determining the levels of inflammation in cells, we found that after HG induction, the contents of pro‐inflammatory TNF‐α, IL‐6, and IL‐1β were elevated and the contents of these cytokines were reduced as a result of Hmgcs2 downregulation (*p* < .01, Figure 3A–C). Meanwhile, we tested the levels of oxidative stress in cells and found that HG induction upregulated ROS and MDA levels and downregulated SOD levels, while Hmgcs2 downregulated led to the opposite changes (*p* < .01, Figure 3D–F). These results suggested that Hmgcs2 downregulation inhibited HG‐induced cell inflammation and oxidative stress.

### 3.4 miR‐363‐5p is the upstream miRNA of Hmgcs2

**Figure 4:**
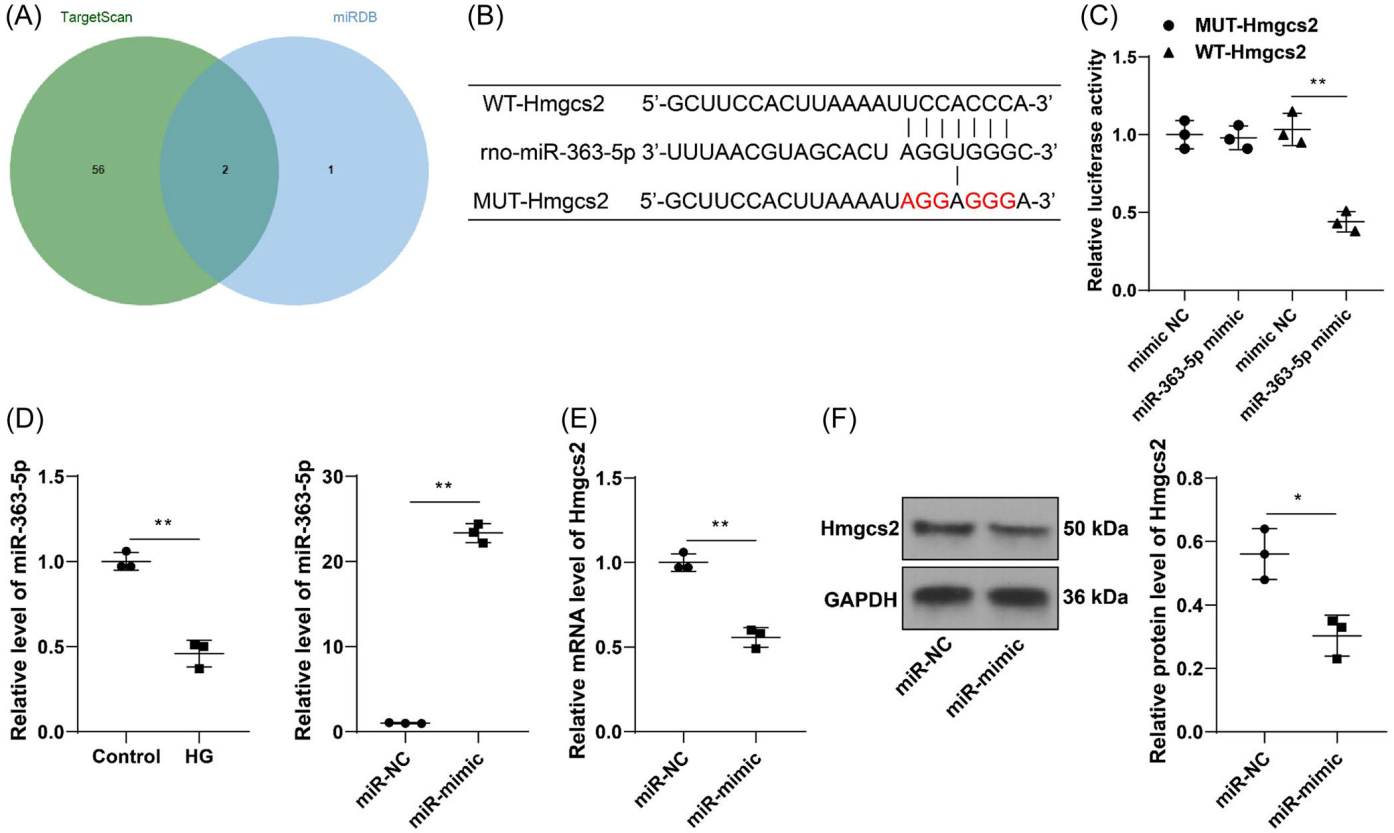
miR‐363‐5p is the upstream miRNA of Hmgcs2. (A) The prediction and intersection of upstream miRNAs of Hmgcs2. (B) Prediction of the binding site of Hmgcs2 and miR‐363‐5p. (C) The targeted binding of Hmgcs2 to miR‐363‐5p was tested by the dual‐luciferase assay. (D) mRNA levels of miR‐363‐5p were determined by RT‐qPCR. (E, F) Expression levels of Hmgcs2 after miR‐363‐5p upregulation were determined by RT‐qPCR and Western blot assay. *N* = 3. Data in panels D, E, and F were analyzed by the *t* test, and data in panel C were analyzed by one‐way ANOVA, followed by Tukey's multiple comparison test. *p < .05, **p < .01.

We further predicted the upstream miRNAs of Hmgcs2 (Figure 4A) and obtained two candidate miRNAs, among which the predicted scores of miR‐363‐5p ranked high in both databases (Supplementary Tables 1 and 2). It suggested that miR‐363‐5p may affect the progression of DCM through regulation of Hmgcs2. According to obtained binding site (Figure 4B), we performed the dual‐luciferase assay and found that cotransfection of WT‐Hmgcs2 and miR‐363‐5p mimics reduced luciferase activity in H9C2 cells (*p* < .01, Figure 4C), which validated the targeted binding of Hmgcs2 to miR‐363‐5p. Additionally, a decrease in the mRNA levels of miR‐363‐5p was observed in HG‐induced cells (*p* < .01, Figure 4D). Then, we upregulated intracellular expression of miR‐363‐5p (*p* < .01, Figure 4D), upon which Hmgcs2 expression levels were significantly downregulated (*p* < .05, Figure 4E,F). Collectively, miR‐363‐5p was found to be the upstream miRNA of Hmgcs2.

### 3.5 miR‐363‐5p overexpression attenuates HG‐induced cell injury

**Figure 5:**
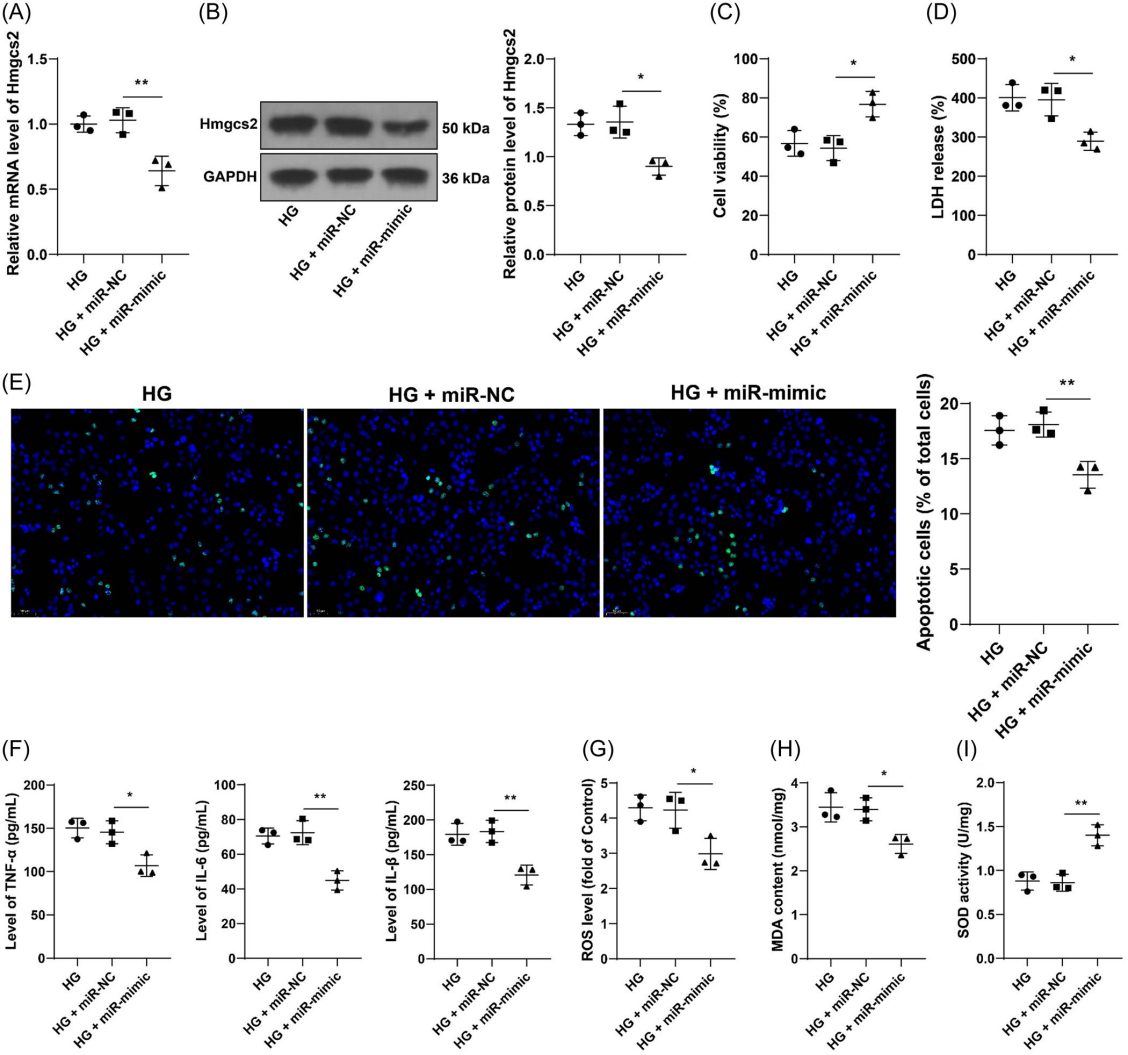
miR‐363‐5p downregulation attenuates HG‐induced cell injury. H9C2 cells were transfected with miR‐363‐5p mimic (miR‐mimic), with cells transfected with miR‐NC as the negative control, followed by HG induction. (A, B) Expression levels of Hmgcs2 were determined by RT‐qPCR and Western blot assay. (C) Cell viability was tested by MTT assay. (D) LDH release of each cell group. (E) Cell apoptosis was evaluated by TUNEL assay (scale bar = 50 μm). (F) Contents of TNF‐α, IL‐6, and IL‐1β were determined by ELISA; G: ROS levels of each cell group. (H) MDA levels of each cell group. (I) SOD levels of each cell group. *N* = 3. Data in panels A–I were analyzed by one‐way ANOVA, followed by Tukey's multiple comparison test. *p < .05, **p < .01.

At last, intracellular expression of miR‐363‐5p was upregulated (*p* < .01, Figure 4D), and cells with miR‐363‐5p upregulation were stimulated by HG. After miR‐363‐5p overexpression, Hmgcs2 expression levels were downregulated (*p* < .05, Figure 5A,B). Overexpression treatment of miR‐363‐5p increased cell viability (*p* < .05, Figure 5C), and reduced LDH release and apoptosis rate (*p* < .01, Figure 5D, E). In addition, the contents of TNF‐α, IL‐6, and IL‐1β were markedly decreased (*p* < .05, Figure 5F), ROS and MDA levels were reduced, while SOD levels were elevated (*p* < .05, Figure 5G‐I) in response to miR‐363‐5p overexpression. These findings indicated that miR‐363‐5p downregulation plays an alleviative role in HG‐induced cell injury.

## 4 DISCUSSION

Diabetic cardiomyopathy (DCM) is a unique form of DM complication that increases the risk of heart failure and causes high mortality.^27^ However, the molecular mechanism that leads to DCM progression remains elusive. High‐throughput sequencing technology has great advantage to screen genes and molecules related to pathophysiology of DCM. In the current study, microarray datasets, including GSE6880 and GSE5606, and a map of gene interaction network revealed that Hmgcs2 plays the most crucial regulatory role in DCM among all DEGs and relevant cellular experiments validated that miR‐363‐5p inhibits Hmgcs2 expression, thereby alleviating HG‐induced cardiomyocyte injury.

NThe GSE6880 and GSE5606 chips have been applied to screen mitochondrial DEnGs and uncovered 9 hub genes related to mitochondrial metabolism and immune microenvironment in DCM.^28^ The GSE5606 chip has also uncovered ANGPTL4 as the most vital hub gene in DCM that promotes cardiomyocyte apoptosis.^29^ In this study, we used GSE6880 and GSE5606 chips to screen DEGs in DCM and identified 23 common DEGs including Hmgcs2. Gene interaction analysis further showed that Hmgcs2 had the most extensive connectivity throughout the network, and therefore may play the most significant role in DCM progression over other DEGs. Hmgcs2 is a vital enzyme that mediates ketogenesis.^11^ High‐fat diet can potentiate ketogenesis and increase Hmgcs2 level in the heart, suggesting that obesity is a risk factor that induces Hmgcs2 upregulation in DCM.^30^ Exercise‐induced Hmgcs2 downregulation can prevent lipotoxicity‐induced cardiomyopathy,^31^ and the activation of PDK4/Hmgcs2 axis is correlated with HG‐induced myocardial injury,^13^ suggesting that Hmgcs2 mediates cardiac dysfunction both in hyperglycemic or non‐hyperglycemic conditions. By virtue of prior evidence and our data, we selected Hmgcs2 for the subsequent cellular assay to validate its role.

A plethora of studies have provided evidence that HG activates maladaptive signaling pathways that lead to apoptosis, cytotoxicity, inflammatory response cascades, and oxidative burst.^32^, ^33^, ^34^ Alleviation of these pathogenic traits can improve ventricular remodeling and cardiac function in the DCM rat model.^35^, ^36^ Hmgcs2 is a key factor associated with inflammation and oxidative injury. For instance, low Hmgcs2 correlates with inflammatory responses in inflammatory bowel disease^37^; the release of Hmgcs2 involves the mechanism of the nonsteroidal anti‐inflammatory drug Celecoxib concerning mitochondrial protection^38^; low Hmgcs2 intensifies oxidative stress in hypertensive rats through less phosphorylation of Akt.^39^ In accordance, our findings revealed that HG treatment decreased cell viability, and increased apoptosis, cytotoxicity (LDH), pro‐inflammatory cytokines (TNF‐α, IL‐6, IL‐1β), and oxidative stress (high ROS/MDA, low SOD). Then, the downregulation of Hmgcs2 reversed these HG‐induced changes in H9C2 cells. Likewise, a very recent study also has demonstrated that inhibition of Hmgcs2 leads to increased viability and reduced apoptosis, inflammation, and oxidative injury in HG‐induced cardiomyocytes.^5^ However, this study lacks analysis of the reason for Hmgcs2 upregulation in DCM. Our study further predicted the upstream mechanism of Hmgcs2 and validated miR‐363‐5p, a much less studied miRNA, as the upstream target of Hmgcs2 in DCM.

The upstream regulation of Hmgcs2 is associated with acetylation, methylation, transcription factors, miRNAs.^40^, ^41^, ^42^, ^43^ Nevertheless, there is extremely limited knowledge of the regulatory relationship between miRNAs and Hmgcs2. A number of miRNAs have deem demonstrated to regulate the progression of DCM. For example, miR‐30c overexpression exerted protective roles in DCM by elevating glucose mechanism, reducing ROS generation, lipid accumulation, and cardiomyocyte apoptosis^44^; inhibition of miR‐150‐5p promoted cardiomyocyte ferroptosis and the development of DCM^45^; a randomized controlled trial suggested that miRNA‐21 is associated with mitochondrial biogenesis and cardiomyocyte apoptosis, with high diagnostic efficiency in subclinical DCM.^46^ Therefore, we predicted the upstream miRNAs of Hmgcs2 through databases, and miR‐363‐5p was found to have the higher prediction score and as a result was selected to analyze its association with Hmgcs2 and its role in DCM. miR‐363 is a predictive, diagnostic, and prognostic biomarker for type 2 diabetes.^47^ In addition, miR‐363 is able to suppress the pro‑inflammatory cytokine levels in cerebral ischemia/reperfusion injury^48^ and moderate oxidative stress in the context of propofol‐induced neurotoxicity.^49^ Our experimentation suggested miR‐363‐5p was downregulated in HG‐induced H9C2 cells and negatively regulated Hmgcs2, and miR‐363‐5p overexpression attenuated HG‐induced cell injury as evidenced by decreased LDH release, apoptosis, inflammation, and oxidative stress. Collectively, our findings suggested that miR‐363‐5p may protect against DCM by repressing Hmgcs2. However, we only validated our mechanism at the cellular level, lacking in vivo validation and application to the clinical study. In addition, there are many upstream miRNAs of Hmgcs2 and the potential competitive endogenous RNA mechanism involving miR‐363‐5p is yet to be studied. With future endeavors, more complementary experiments are needed to explore and improve the integrity of Hmgcs2‐mediated regulatory network in DCM, and animal experiments shall be conducted to validate our mechanism.

## 5 CONCLUSION

To summarize, our study uncovered that Hmgcs2 is the vital hub gene in the regulatory network of DCM and for the first time reported that miR‐363‐5p negatively regulates Hmgcs2 to play an alleviative role in HG‐induced cardiomyocyte injury, which may retard the progression of DCM. Our findings suggested that Hmgcs2 and miR‐363‐5p may be targets for the diagnosis and management of DCM.

## AUTHOR CONTRIBUTIONS

**Ying Wang**: Experimental studies; manuscript preparation; manuscript editing. **Li‐Feng Ping**: Data acquisition; data analysis. **Fu‐Yan Bai**: Clinical studies; statistical analysis. **Xin‐Huan Zhang**: Study concepts; definition of intellectual content. **Guang‐Hong Li**: Guarantor of integrity of the entire study; study design.

## CONFLICTS OF INTEREST STATEMENT

All authors declare that there is no conflict of interests in this study.

## Supporting information

Supporting information.

Supporting information.

## Data Availability

The data that support this study are available from the corresponding author upon reasonable request.
